# Remote ischemic conditioning for the treatment of ischemic moyamoya disease

**DOI:** 10.1111/cns.13279

**Published:** 2019-12-08

**Authors:** Jia‐yue Ding, Shu‐ling Shang, Zhi‐shan Sun, Karam Asmaro, Wei‐li Li, Qi Yang, Yu‐chuan Ding, Xun‐ming Ji, Ran Meng

**Affiliations:** ^1^ Department of Neurology Xuanwu Hospital Capital Medical University Beijing China; ^2^ Advanced Center of Stroke Beijing Institute for Brain Disorders Beijing China; ^3^ Department of China‐America Institute of Neuroscience Xuanwu Hospital Capital Medical University Beijing China; ^4^ Tangshan Union Medical College Hospital Tangshan China; ^5^ Department of Neurosurgery Weifang People's Hospital Wenfang China; ^6^ Department of Neurosurgery Wayne State University School of Medicine Detroit MI USA; ^7^ Department of Neurosurgery Henry Ford Health System Detroit MI USA; ^8^ Department of Neurosurgery Xuanwu Hospital Capital Medical University Beijing China

**Keywords:** moyamoya disease, remote ischemic conditioning, stroke, treatment

## Abstract

**Aims:**

This study investigated the safety and efficacy of remote ischemic conditioning (RIC) on ameliorating the sequelae of ischemic moyamoya disease (iMMD).

**Methods:**

A total of 30 iMMD patients underwent long‐term RIC and were followed up at 0.5, 1, and 2 years for clinical outcomes, including frequency of stroke recurrence, Patient Global Impression of Change (PGIC) scale, peak systolic velocities (PSV), and cerebral perfusion.

**Results:**

During the whole RIC treatment process, no RIC‐related adverse event occurred. Only one of 30 patients suffered a onetime infarction (3.3%), and the ratios of acceptable PGIC were 88.2%, 64.3%, and 92.3% at 0.5, 1, and 2 years follow‐up. Kaplan‐Meier analysis showed the frequency of stroke recurrence was significantly reduced after RIC (*P* = .013). The frequency of TIA per week was 1.1 (0.6, 2.8) prior to RIC and 0.1 (0.0, 0.5) post‐RIC (*P* < .01). Compared to baseline, PSV values were significantly reduced after RIC treatment (*P* = .002 at 0.5, *P* = .331 at 1, and *P* = .006 at 2 years). In patients undergoing perfusion studies, 75% obtained improvement on followed‐up SPECT and 95% on followed‐up PET maps.

**Conclusions:**

Remote ischemic conditioning may be beneficial on controlling iMMD‐induced ischemic events, relieving symptoms, and improving cerebral perfusion, without incidence of complications in this case series.

## 1 INTRODUCTION

Moyamoya disease (MMD) is a progressive steno‐occlusive disease of the distal internal cerebral arteries, and its immediate branches lead to compensatory development of arterial vasculature, which are fragile, small, and prone to frequent thrombosis and hemorrhages.^1^ Current therapy demands for revascularization surgery either by direct, indirect, or combined techniques to improve cerebral blood flow (CBF) and decrease the incidence of stroke.^2^ Although refined with modern microsurgical techniques, surgical treatment is not without risk and potential complications.^3^ Hyperperfusion syndrome, described in 21%‐50% of MMD revascularization patients, can occur from sudden changes in cerebral hemodynamics and places patients at a risk for perioperative hemorrhage and postoperative neurological deficits.^4^, ^5^ Considered one of the most technically demanding procedures in neurosurgery, inadequate training and insufficient experience in bypass surgery of MMD—due to the inherent paucity of the disease in most clinical settings—can lead to increased rates of operative failure and morbidity, resulting in disability and even death.^3^ Thereby, more safe and effective treatment strategies of MMD are warranted.

Recently, remote ischemic conditioning (RIC) has garnered great attention due to its efficacious therapeutic benefit on atherosclerotic intracranial arterial stenosis (ICAS).^6^, ^7^, ^8^ It has been demonstrated that RIC can increase the tolerance to cerebral ischemia, reduce stroke recurrence, improve cerebral perfusion, and promote compensatory collateral network formation.^9^, ^10^, ^11^ Moreover, RIC is a noninvasive, easy‐to‐use, and low‐cost strategy in comparison with revascularization surgery.^6^, ^7^, ^8^ Herein, we hypothesized that RIC may yield a safe and effective treatment strategy to patients with ischemic MMD (iMMD), a modality that is hitherto undescribed for this disease entity.

## 2 METHODS

### 2.1 Subjects population

This single‐arm, open‐label, safety, and feasibility study had been approved by the institutional ethic committee of Xuanwu Hospital, Capital Medical University (Beijing, China), in accordance with the guidelines of the 1964 Declaration of Helsinki. Informed consents were obtained from all patients before initiating any study‐specific procedures. From July 2008 through July 2016, a total of 30 patients with confirmed iMMD were recruited into this study. All of the iMMD patients enrolled matched the criteria of diagnosis declared by the Research Committee of MMD of the Japanese Ministry of Health, Labor, and Welfare in 2015.^12^, ^13^

**Figure 1:**
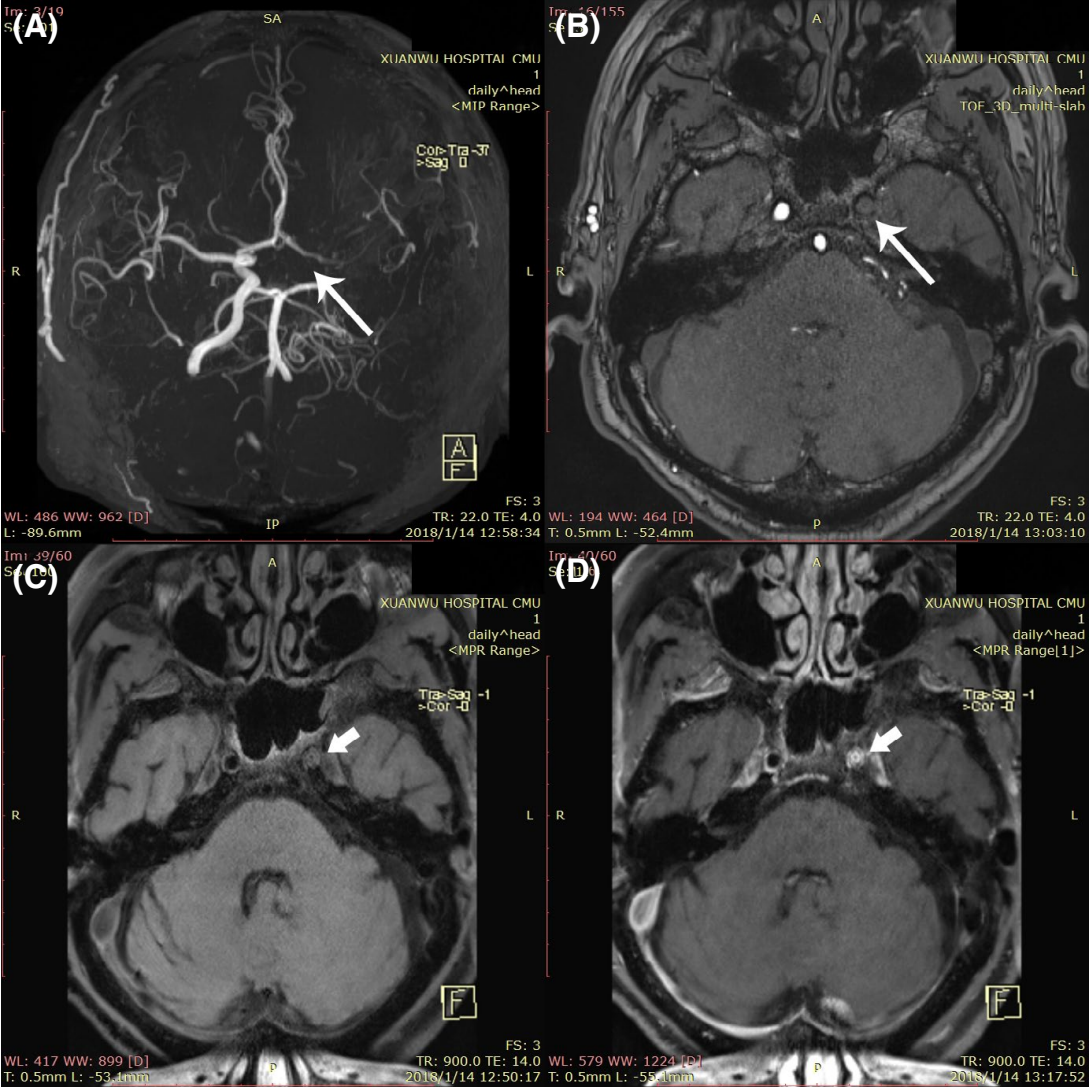
Features of moyamoya vessels on TOF MRA and HR‐MRI map. TOF MRA shows (A) cross‐sectional image showed moyamoya vessel, (B) the left internal carotid artery with complete occlusion (long arrow), (C) T1‐weighted HR‐MRI, and (D) contrast‐enhanced HR‐MRI present the concentric inward remodeling (short arrow) in the left internal carotid artery lumen

Inclusion criteria included computed tomography angiography (CTA) or magnetic resonance angiography (MRA) showing (a) steno‐occlusive disease existing in the uni‐ or bilateral terminal internal carotid arteries (ICA), proximal anterior cerebral arteries (ACA), and/or middle cerebral arteries (MCA); (b) numerous “puff‐of smoke” abnormal vascular networks grown at the base of the brain; (c) high‐resolution magnetic resonance imaging (HR‐MRI) was conducted in some patients with indefinite MMD. Concentric narrowing of the vessel lumen without plaque or eccentric wall thickening on HR‐MRI was considered as early morphological changes of MMD (Figure 1); and (d) enrolled patients with only ischemic attacks were enrolled, excluding ones with hemorrhagic events.

Exclusion criteria involved (a) patients afflicted by other austere life‐threatening diseases; (b) patients with poor compliance; and (c) patients with a history of bypass surgery.

### 2.2 Interventions and assessment

All of the iMMD patients enrolled underwent bilateral upper limb RIC intervention three times daily, consisting of five cycles alternating between 5 minutes of ischemia (induced by inflating tourniquets with the pressure of 200 mm Hg) and 5 minutes reperfusion (inflating pressure was 0 mm Hg).^10^ The RIC was achieved through an electric auto‐control device that has been delineated in our previous study (patent number ZL200820123637.X, China).^10^ Meanwhile, routine treatment, such as antiplatelet therapy, was as the same as the traditional medical treatment prior to RIC.

The frequency of ischemic events, Patient Global Impression of Change (PGIC) scales, peak systolic velocities (PSV) on transcranial Doppler (TCD), arterial morphology on time‐of‐flight MRA (TOF MRA), and cerebral perfusion evaluated by magnetic resonance perfusion weighted imaging (MR‐PWI), single‐photon emission computed tomography (SPECT), and positron emission computed tomography (PET) were all recorded.

Transcranial Doppler with a 2‐MHz probe was used to detect cerebral arterial blood flow through the temporal and suboccipital windows. Magnetic resonance images (MRI) as well as MRA and PWI were conducted on a 3T system (MAGNETOM Verio, Siemens Healthcare, Erlangen, Germany) with a 32‐channel head coil for signal reception at baseline and 1 month, 3 months, 6 months, 1 year, and 2 years of follow‐up after initiating RIC. Modified Suzuki scoring was used to evaluate the severity of disease (seen in Table S1).^1^ Regional cerebral blood flow was assessed with technetium‐99m ethylene cysteine dimer (^99m^Tc‐ECD) on SPECT and ^13^N‐Ammonia on PET. SPECT scan was conducted at the 30th minute after IV ^99m^Tc‐ECD (25 mCi) bolus injection, and PET scan was performed at the 5th minute after IV ^13^N‐ammonia (8 mCi) bolus injection. Original images were reconstructed as perfusion maps in a 128 × 128 matrix and presented in coronal, axial, and sagittal planes by the Butterworth filtering function.

### 2.3 Outcome measurements

Safety‐related outcome endpoints consisted of signs of tissue or vascular injury such as local edema, erythema, skin lesions, allergy, or intolerance for RIC. Other suspected events related to RIC were also recorded.

The incidence of recurrent ischemic events and overall treatment responses on the PGIC scales at follow‐up were regarded as the primary outcomes. Recurrent ischemic events during RIC treatment included stroke and transient ischemic attacks (TIA). Stroke recurrence referred to a new cerebral infarct or hemorrhagic lesions as detected by diffusion‐weighted image (DWI) or fluid attenuated inversion recovery (FLAIR) weighted sequences, respectively. TIA events were defined as neurological symptoms and signs resolving spontaneously within 24 hours after symptoms onset without evidence of infarction on MRI. The incidence of recurrent stroke prior to undergoing RIC was compared with that during RIC intervention by Kaplan‐Meier analysis. The PGIC is a self‐reported evaluation of symptoms by the patients in the study; it consists of a 5‐point scale (1 = significant reduction or elimination of symptoms; 2 = mild attenuation; 3 = no change; 4 = mild deterioration; 5 = significant deterioration).^14^ Radiographic findings and the changes in overall symptomatology were recorded at outpatient follow‐up.

Secondary outcomes included frequency of TIA, PSV on TCD, perfusion status on SPECT, PET or PWI, modified Rankin scale (mRS) scores, and National Institute of Health stroke scale (NIHSS) scores. The measurement aforementioned at follow‐up was compared with those at admission in order to uncover the effectiveness of RIC on patients with iMMD. According to the diagnosis criteria of cerebral arterial stenosis via TCD, PSV of >120 cm/s in ACA and MCA were considered abnormal, reflecting on stenosis seen on MRA or CTA.^15^ Based on the signal intensity presented by the SPECT map, perfusion status was categorized into six intensity scales (0 = 0%, 1 = 0%‐25%, 2 = 25%‐50%, 3 = 50%‐75%, 4 = 75%‐100%, and 5 = 100%). The arterial territories comprised of the bilateral frontal, parietal, occipital, and temporal lobes; basal ganglia; and cerebellum. The difference of the total signal intensities pre‐ and post‐RIC was assessed. Furthermore, the overall perfusion improvement and deterioration rates were also recorded. The definition of perfusion improvement referred to the total signal intensity scale increase by two or more and deterioration referred to the total signal intensity scale decrease by two or less. The perfusion status on PET map was evaluated by the ratio of impaired lobe vs cerebellum perfusion that was presented as the ^13^N‐ammonia uptake index.

### 2.4 Statistical analysis

SPSS 19.0 was used for analysis in this study. Continuous data following a Gaussian distribution were presented as mean ± standard deviation and analyzed with Student's *t* test; otherwise, the data were presented as median (interquartile range, IQR) and analyzed with a Mann‐Whitney *U* test. Categorical data were expressed as n (%) and were processed by chi‐square test (for dichotomous variable) or Mann‐Whitney *U* test (for ordinal dependent variable). Kaplan‐Meier curve and log‐rank test (Mantel‐Cox) were used to compare the stroke recurrence frequency between pre‐ and post‐RIC treatment; *P* < .05 was indicative of statistical significance.

## 3 RESULTS

### 3.1 Population characteristics

A total of 30 patients were enrolled (12 adult and 18 children), in which bilateral MMD accounted for 90% (27/30), while the remaining were unilateral MMD (10%), the average age was 22.0 ± 17.6 years. There were 18 (60%) patients who presented with a TIA, the remainder of patients presented with infarctions, and all the events were confirmed by MRI. The top two clinical manifestations included paralysis (63.3%) and headache (30%). The average time from their symptom‐onset to enrollment was 6.0 months (IQR: 3.5‐23.2), and the average follow‐up time after RIC treatment was 16.8 months (IQR: 6.8, 24.0). Six (20%) patients suffered comorbid diseases (including hypertension, diabetes, or hyperlipemia), and all of the iMMD diagnoses in these six patients were confirmed by HR‐MRI to rule‐out intracranial atherosclerotic disease as the cause of stenosis (Figure 1), and details are shown in Table 1.

**Table 1:** Baseline characteristics of MMD patients treated with RIC

Characteristics	Number of patients (n = 30)	(%)
Demographics		
Female	12	40.0
Male	18	60.0
Age, years	22.0 ± 17.6	NA
Adult	12	40.0
Child	18	60.0
Smoke	2	6.7
Drink	2	6.7
Clinical manifestations		
Time from symptom onset to enrollment, months	6.0 (3.5, 23.2)	NA
Follow‐up time	16.8 (6.8, 24.0)	NA
TIA	18	60.0
Headache	9	30.0
Dizziness	2	6.7
Paralysis	19	63.3
Paresthesia	3	10.0
Visual disorder	3	10.0
Aphasia	4	13.3
Comorbid disease		
Hypertension	4	13.3
Diabetes	2	6.7
Hyperlipemia	2	6.7
Comorbid disease free	24	80.0
Imaging findings		
Unilateral stenosis	3	10.0
Bilateral stenosis	27	90.0
Stenosis position		
Left distal ICA	16	53.3
Right distal ICA	16	53.3
Left MCA	24	80.0
Right MCA	26	86.7
Left ACA	15	50.0
Right ACA	12	40.0
Left PCA	4	13.3
Right PCA	3	10.0
Modified Suzuki scoring		
Stage I	6	20.0
Stage II	6	20.0
Stage III	9	30.0
Stage IV	9	30.0
Brain tissue infarction	12	40.0

Note

Continuous variates following a Gaussian distribution were presented as mean ± standard deviation; otherwise, the vairates were presented as median (interquartile range, IQR). Categorical data were expressed as n (%).

### 3.2 Safety outcomes

No RIC‐related adverse events occurred during RIC intervention. All patients in this study tolerated to RIC strategy and completed the whole study. No major discomfort as a result of RIC treatment was reported.

### 3.3 Primary outcomes

**Figure 2:**
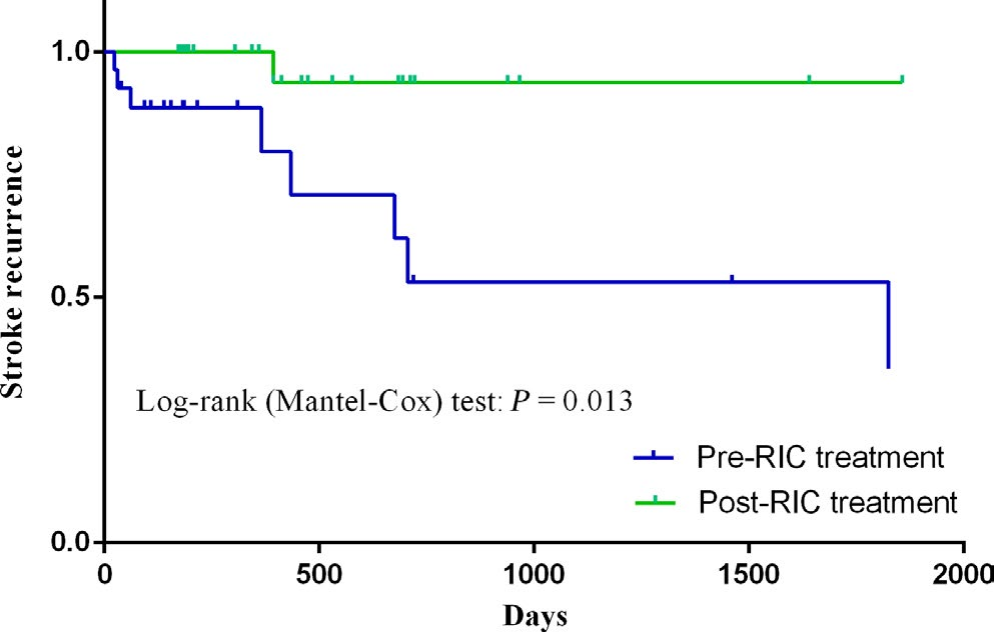
Kaplan‐Meier curve showing the frequency of stroke recurrence pre‐ and post‐RIC. The incidence of recurrent stroke after RIC was significantly reduced compared to before RIC (log‐rank [Mantel‐Cox] test: *P* = .013)

One (3.3%) patient reported having a new infarction at follow‐up. A total of 17 patients who finished 6 months of follow‐up had no stroke recurrence. In terms of PGIC, 15 patients (88.2%) reported that their symptoms were significantly attenuated or disappeared, while 2 (11.8%) had mildly attenuation of symptoms. Fourteen patients finished 1‐year follow up, in whom a newly ischemic brain lesions were found on FLAIR‐weighted image of one patient (7.1%). In regard to PGIC, 9 of the 14 patients (64.3%) reported significant attenuation or elimination of symptoms, 4 (28.6%) had mild attenuation, and one patient (7.1%) complained of mild deterioration. Thirteen patients finished 2‐year follow up, no recurrent stroke was reported in 12 patients (92.3%), while 1 (7.7%) was found to have a similar ischemic lesion that has been recorded at 1‐year follow up, aforementioned above; 12 of the 13 cases (92.3%) reported significant attenuation or elimination and 1 (7.7%) had mild attenuation. Only four patients were available for 3‐year follow‐up, two patients finished 4‐year follow‐up, and one patient finished 5‐year follow‐up; the symptoms of these patients disappeared completely, and no recurrent strokes were recorded. No cerebral hemorrhage events occurred in all the 30 patients during the whole process of RIC intervention. Table 2 shows the complete data entries. Subgroup analysis presented in support information (Table S2‐S5) indicated that there was no difference in primary outcomes when stratified from symptom onset to treatment time (<1 year and ≥1 year) and modified Suziki scores (stage I‐IV). Twenty‐seven of the 30 patients underwent traditional medication treatment prior to RIC, of which 8 cases suffered ischemic stroke recurrence during that period. When compared to pre‐RIC treatment, the incidence of stroke after RIC was significantly decreased (29.6% vs 3.7%, *P* = .024). The frequency of stroke recurrence prior to and post‐RIC in all 27 patients was analyzed with Kaplan‐Meier analysis (Figure 2), which showed a significant recurrent stroke frequency reduction after RIC, log‐rank test (Mantel‐Cox) *P* = .013. Twelve patients manifested with TIA. The frequencies of TIA per week were 1.1 (range: 0.6‐2.8) prior to RIC and 0.1 (range: 0‐0.5) after RIC (*P* < .01).

**Table 2:** Primary outcomes for MMD patients treated with RIC

	Number of patients	Incidence of stroke recurrence (%)	Much improvement (%)
6‐month follow‐up (190 ± 25 d)	17	0 (0.0)	15 (88.2)
1‐year follow‐up (356 ± 65 d)	14	1 (7.1)	9 (64.3)
2‐year follow‐up (663 ± 73 d)	13	1 (7.7)^a^	12 (92.3)
3‐year follow‐up (963 ± 18 d)	4	0 (0.0)	4 (100)
4‐year follow‐up (1583 ± 81 d)	2	0 (0.0)	2 (100)
5‐year follow‐up (1857 d)	1	0 (0.0)	1 (100)

Note

Of the 30 involved patients, only one (3.3%) subject suffered brain infarction one time during the overall follow‐up.

a The recurrent stroke event occurred 1 y ago.

### 3.4 Neurological dysfunction and arterial PSV in TCD

The NIHSS and mRS scores were used to evaluate permanent neurological dysfunction. The NIHSS and mRS scores at baseline were 0 (range: 0‐1) for both. After RIC treatment, fluctuation of scores at all follow‐up time points showed no statistical difference.

The PSV in the MCA and ACA vasculature greater than 120 cm/s was considered abnormal to compensate for increases in velocity secondary to vasculature stenosis. For arteries with initial abnormal PSV prior to RIC, the average PSV at 6 months of RIC (nine patients, 15 vessels) was significantly lower than baseline (193.1 ± 88.8 cm/s vs 248.8 ± 80.5 cm/s, *P* = .022); the average PSV at 1 year of RIC (11 patients, 16 vessels) vs baseline was not statistically different (195.2 ± 74.0 cm/s vs 210.5 ± 45.0 cm/s, *P* = .331); and the average PSV at 2 years of RIC (seven patients, 11 vessels) was significantly lower than that of baseline (135.2 ± 87.8 cm/s vs 245.3 ± 71.0 cm/s, *P* = .006). However, with respect to arteries with normal PSV at baseline, there were two vessels (from two patients) with elevated PSV after 1 year of RIC.

### 3.5 Imaging presentations at follow‐up

**Figure 3:**
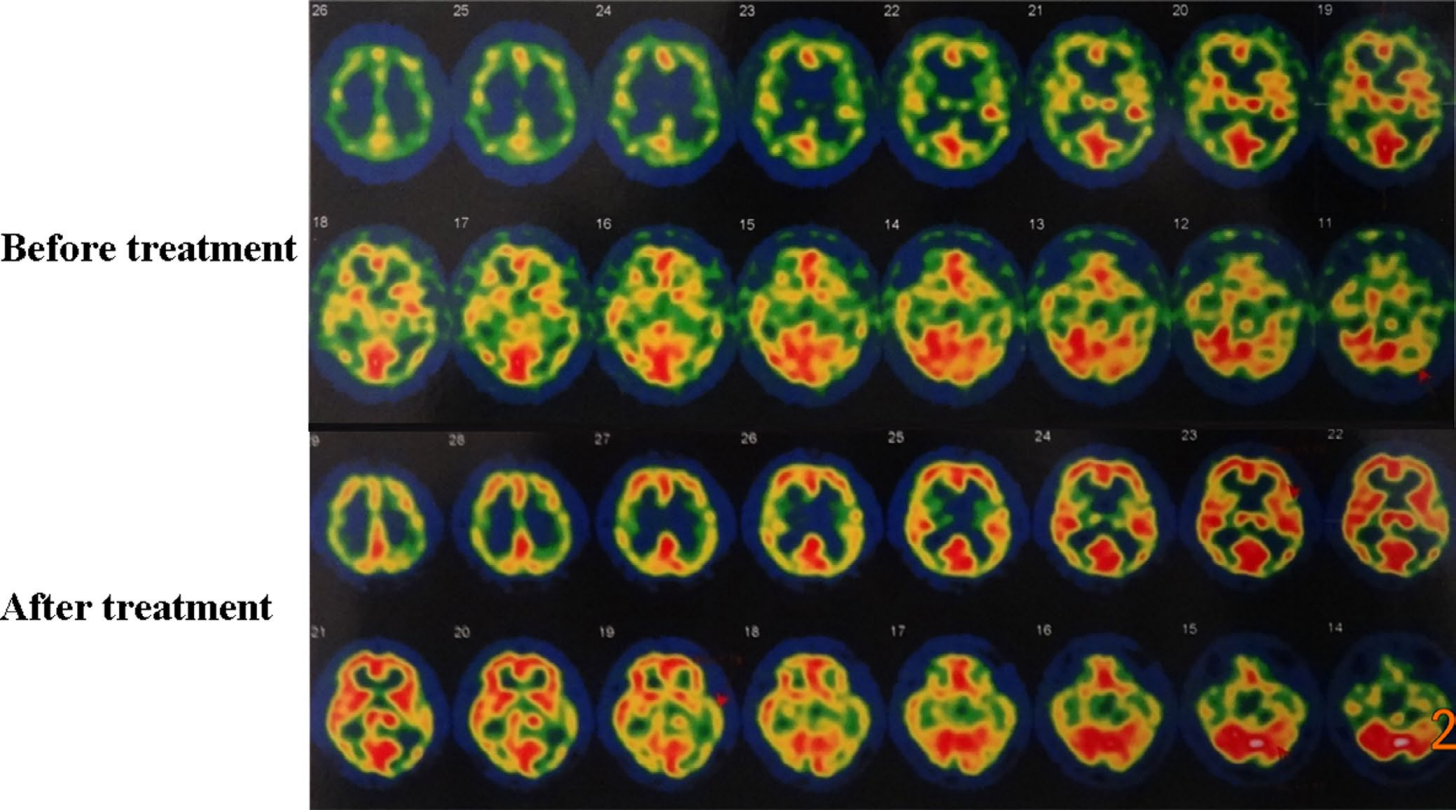
Perfusion presented on SPECT maps prior to and post‐RIC treatment. SPECT shows that the perfusion decreases in bilateral frontal, parietal, occipital, and temporal lobes before RIC regimen. After 1 year of RIC treatment, the perfusion is significantly enhanced in all lobes

A total of nine patients underwent SPECT pre‐ and post‐RIC, eight of which finished 1‐year follow‐up, 6 (75%) had perfusion improvement, 1 case showed no remarkable perfusion improvement, and 1 showed local perfusion decline after RIC. It should be noted that the declined perfusion in the last patient at 1 year improved remarkably at the 4‐year follow‐up, which was better than both baseline and 1 year post‐RIC. Quantitative analysis of signal intensity also showed profoundly increased perfusion after RIC (pre‐ vs post‐RIC: 32.6 ± 5.1 vs 38.3 ± 3.0, *P* = .036). One patient, however, finished 2 years of RIC treatment without perfusion improvement. Typical perfusion status pre‐ and post‐RIC is shown in Figure 3.

Twenty patients finished PET evaluation at both pre‐ and post‐RIC. The results showed that the perfusion was improved in 19 cases after RIC, except for 1 case of perfusion deteriorated. Quantitative data from five patients analyzed showed a tendency of perfusion mismatch attenuated in moyamoya vessels territory after RIC (pre‐ vs post‐RIC: 0.757 vs 0.843, *P* = .162).

**Figure 4:**
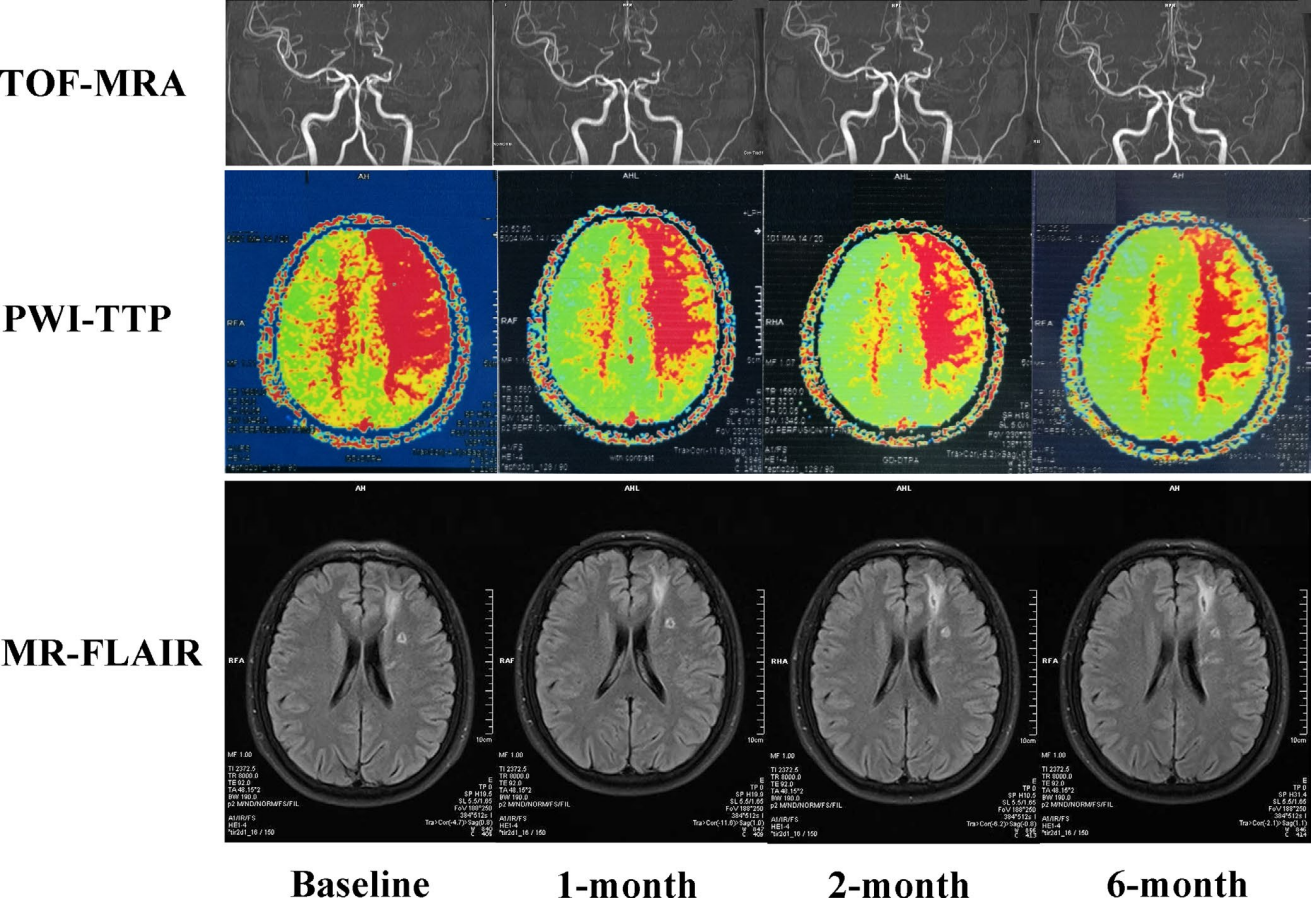
Dynamic changes of MR images of a case at 6 months post‐RIC. A 35‐year‐old male MMD patient with left distal MCA stenosis (TOF MRA) and left frontal infraction (MR‐FLAIR) at baseline prior to RIC and after 1, 2, and 6 months of RIC. PWI showed the degree of prolonged TTP in left frontal and parietal lobe were remarkably attenuated after he underwent 1, 2, and 6 months of RIC, which meant that the collateral circulation formed to shorten the blood transit time TTP values. In the 1, 2, and 6 months of RIC follow‐up time points, there was no recurrent stroke shown on MR‐FLAIR and no progressive stenosis presented on TOF MRA

One case underwent dynamic MR PWI prior to and post‐RIC evaluation. The elevated time‐to‐peak (TTP) value was significantly shortened after RIC at 1, 2, and 6 months. During this period, no newly stroke lesions were observed on FLAIR weighted images and the arterial status appeared stable (Figure 4).

## 4 DISCUSSION

The steno‐occlusion of the circle of Willis leads to a reduction of CBF in the territory irrigated by the affected vessels. As a result, compensatory collateral networks depicted as a “puff‐of smoke” from the deep thalamoperforating and lenticulostriate perforating arteries are formed extensively in an attempt to maintain adequate CBF. The smoke‐like vessels are fragile and thin, resulting in thrombosis formation and microaneurysm rupture, leading to cerebral ischemia and hemorrhage.^12^, ^16^ Furthermore, lack of adequate blood flow can lead to cortical and subcortical ischemia, resulting in neurological deficits.^17^, ^18^, ^19^, ^20^ Hence, improvement and restoration of CBF in moyamoya is a topic of ongoing interest.

Currently, revascularization surgery (direct or indirect techniques) is considered as the standard method to MMD, where options are limited and other strategies have proven to be futile. However, the risks of revascularization surgery are not eliminated, even in experienced hands; the rate of morbidity ranges from 3.5% to 4%, and death has been estimated to be at 0.7% per treated hemisphere.^21^, ^22^, ^23^, ^24^, ^25^, ^26^ A recent meta‐analysis declared that bypass surgery significantly reduces stroke events compared with conservative treatment for iMMD.^21^ Meanwhile, some studies have also reported a lack of clear benefit in the surgical arm for the treatment of iMMD.^22^, ^23^ Furthermore, bypass surgery in China costs approximately 30 000 USD or more. Whereby, if a noninvasive strategy is applied in iMMD patients, it can help patients who are not surgical candidates or can even serve as an adjunct to surgery.

Remote ischemic conditioning strategy has been suggested to treat both acute ischemic stroke and intracranial atherosclerosis (ICAS).^6^, ^7^, ^8^, ^9^, ^10^, ^11^ Our previous study indicated that RIC reduced the incidence of stroke recurrence in ICAS patients from 26.7% to 7.9% after 300 days continuous intervention.^10^ Likewise, another study also verified the safety and efficacy of RIC on patients with ICAS.^11^ As the abnormality of hemodynamics in both ICAS and iMMD stems from large artery stenosis, thereby we hypothesized that RIC can also be effective to patients with iMMD. To the best of our knowledge, this is the first report studying the efficacy and safety of long‐term RIC in MMD.

The primary outcomes in this study demonstrated that the incidence of iMMD–induced stroke recurrence and TIA events after long‐term RIC was significantly lower than that prior to RIC in the same cohort of patients. Furthermore, almost all patients reported that their symptoms were ameliorated after RIC treatment. Chronic cerebral circulation insufficiency in iMMD always causes nonfocal neurological deficits such as headache, dizziness, and insomnia; as a result, the subjective feelings from patients are of great importance. Therefore, PGIC scales, which reflected the emotional and patient‐subjective status, were used as the indicator for symptom amelioration in this study.^14^, ^19^, ^27^ Of the 30 patients, 29 noticed a benefit from RIC, while one patient experienced a minor stroke recurrence and his symptoms mildly fluctuated during the follow‐up period. However, after long‐term continuous RIC, his symptoms were started to wane and the perfusion status significantly improved. In conclusion, at the end of follow‐up, the iMMD processes of all patients enrolled were controlled by long‐term RIC.

Given the relatively mild neurological status at baseline, improvement of function after RIC could not be presented exclusively by NIHSS or mRS scores. Nevertheless, no difference was observed between baseline and the various follow‐up time points after long terms of RIC; this is exactly the indication that RIC may inhibit severe disabling events. PSV data suggested that RIC alleviated hemodynamic instability, which was demonstrated by significantly decreased PSV after RIC. Moreover, SPECT, PET, and PWI data also indicated that RIC led to improved cerebral perfusion, as both the maps of SPECT (75% cases) and PET (95% cases) showed improved cerebral perfusion after 1 year of continuous RIC, and quantitative analysis also supported perfusion improvement after RIC. TTP value on a PWI map also verified the improvement of perfusion after RIC, as TTP was always regarded as the preclinical sensitive indicator.^28^ RIC may promote the autoregulatory vasodilation and increase oxygen extraction fraction in humans to reduce stroke recurrence rate in chronic cerebral circulation insufficiency.^29^ In addition, collateral compensation may contribute to the perfusion improvement, despite with poor evidences of the mechanisms in this study.^30^ Further demonstration based on hemodynamic tests (cerebral vascular response to hypocapnia) may be assist and to confirm the effect of RIC on hemodynamic improvement.^31^

Taken together, our findings demonstrate for the first time that RIC may be a safe and feasible as well as an easy‐to‐use strategy for iMMD control, with potential clinical significance. In comparison with revascularization surgery, RIC is not only a noninvasive strategy with low rate of complications, but also a cheap approach. The electric autocontrol device costs approximate 400 USD, far lower than the cost of bypass surgery. Thus, RIC is a promising strategy for iMMD treatment and further studies are needed before such conclusions can be made.

There were several limitations in this initial pilot study. First, the sample size was small. In addition, this pilot study is a real‐world single‐armed cohort study, without a real‐time bypass surgery control, even though we compared the incidence of stroke prior to and after RIC, in spite of other studies about MMD, which can serve as references for our results. As for stroke recurrence incidence compared prior to and post‐RIC, the symptom onset to enrollment time [6.0 months (IQR: 3.5‐23.2)] was vastly shorter than the follow‐up time [16.8 months (IQR: 6.8, 24.0)]. Even so, the incidence of stroke recurrence before RIC was significantly higher than that after RIC during the whole follow‐up period. The short observational time before RIC may bias the results toward the null hypothesis. The perfusion status scores on SPECT were recorded according to the signal intensity presented on the map with six scales. The assessment might have not been accurate, and the data could potentially bias the actual perfusion status. Finally, the follow‐up time was still not long enough, with 13 patients completing 2‐year follow‐up, and only one patient completing 5‐year follow‐up, also indicating a high drop‐out rate which can confound patients who were adversely harmed by the procedure. On the other hand, resolution of symptoms and lack of stroke and TIA events recurrence could have given patients a sense of security and encouraged them to stop treatment and return to their daily lives, all while avoiding distant travelling for follow‐up. Nevertheless, long‐term observation still needed to find more reliable results. The perfusion improvement was likely to be a prodromal sign for excellent long‐term outcomes in this cohort.

## 5 CONCLUSIONS

Remote ischemic conditioning may be a promising noninvasive method for iMMD control. It contributed to both symptoms relieving and incidence reducing of iMMD‐mediated stroke recurrence and improved cerebral perfusion status as well as at a low cost and safety. Considering the aforementioned limitations, a multicenter, large sample size, and well‐designed prospective clinical trial is ongoing.

## DISCLOSURE

Jiayue Ding, Shuling Shang, Zhishan Sun, Karam Asmaro, Weili Li, Qi Yang, Yuchuan Ding, Xunming Ji, and Ran Meng declare that they have no conflict of interest with the contents of this article.
