# Supplementary material for: Remote ischemic conditioning for the treatment of ischemic moyamoya disease
Source: CNS Neurosci Ther. 2019 Dec 8;26(5):549–57. doi: 10.1111/cns.13279 (PMC7163773; doi:10.1111/cns.13279)
Supplement: Supplementary file 1 [file CNS-26-549-s001.docx]

**Supporting information**

**Table S1.** Modified Suzuki scoring

| Scores | Description |
| --- | --- |
| Stage 0 | no evidence of vessel disease |
| Stage I | mild-to-moderate stenosis around the carotid bifurcation with absent or slightly developed ICA disease |
| Stage II | severe stenosis around the carotid bifurcation or occlusion of either the proximal ACA or MCA with well-developed ICA Moyamoya disease |
| Stage III | occlusion of both the proximal ACA and MCA with well-developed ICA Moyamoya disease (only a few of either the ACA or MCA branches or both are faintly opacified in antegrade manner through the meshwork of ICA Moyamoya disease) |
| Stage IV | complete occlusion of both the proximal ACA and MCA with an absent or a small amount of ICA Moyamoya disease (without opacification of either the ACA or MCA branches in antegrade manner) |

**Table S2.** Subgroup analysis for 6-month follow-up primary outcomes in patients treated with RIC

| 6-month follow-up (190±25 days) | Num. of patients | Incidence of stroke recurrence (%) | Much improvement (%) |
| --- | --- | --- | --- |
| Time from symptom onset to enrollment |  |  |  |
| ≤1year | 12 | 0 (0.0) | 10 (83.3) |
| >1 year | 5 | 0 (0.0) | 5 (100.0) |
| *p*-value | - | - | 0.485 |
| Modified Suzuki scoring |  |  |  |
| Stage I | 4 | 0 (0.0) | 3 (75.0) |
| Stage II | 4 | 0 (0.0) | 3 (75.0) |
| Stage III | 5 | 0 (0.0) | 5 (100.0) |
| Stage IV | 4 | 0 (0.0) | 4 (100.0) |
| *p*-value | - | - | 0.466 |

**Table S3.** subgroup analysis for 1-year follow-up primary outcomes in patients treated with RIC

| 6-month follow-up (190±25 days) | Num. of patients | Incidence of stroke recurrence (%) | Much improvement (%) |
| --- | --- | --- | --- |
| Time from symptom onset to enrollment |  |  |  |
| ≤1year | 7 | 1 (14.3) | 4 (57.1) |
| >1 year | 7 | 0 (0.0) | 5 (71.4) |
| *p*-value | - | 1.000 | 1.000 |
| Modified Suzuki scoring |  |  |  |
| Stage I | 3 | 0 (0.0) | 0 (0.0) |
| Stage II | 2 | 0 (0.0) | 2 (100.0) |
| Stage III | 4 | 1 (25.0) | 3 (75.0) |
| Stage IV | 5 | 0 (0.0) | 4 (80.0) |
| *p*-value | - | 0.442 | 0.064 |
| Of the 30 involved patients, only one (3.3%) subject suffered brain infarction one time during the overall follow-up. | | | |

**Table S4.** subgroup analysis for 2-year follow-up primary outcomes in patients treated with RIC

| 6-month follow-up (190±25 days) | Num. of patients | Incidence of stroke recurrence (%) | Much improvement (%) |
| --- | --- | --- | --- |
| Time from symptom onset to enrollment |  |  |  |
| ≤1year | 8 | 1 (12.5)^*^ | 7 (87.5) |
| >1 year | 5 | 0 (0.0) | 5 (100.0) |
| *p*-value | - | 1.000 | 1.000 |
| Modified Suzuki scoring |  |  |  |
| Stage I | 3 | 0 (0.0) | 2 (66.7) |
| Stage II | 2 | 0 (0.0) | 2 (100.0) |
| Stage III | 6 | 1 (16.7) | 6 (100.0) |
| Stage IV | 2 | 0 (0.0) | 2 (100.0) |
| *p*-value | - | 0.738 | 0.307 |
| Of the 30 involved patients, only one (3.3%) subject suffered brain infarction one time during the overall follow-up. ^*^The recurrent stroke event occurred 1 year ago. | | | |

**Table S5.** subgroup analysis for ＞3-year follow-up primary outcomes in patients treated with RIC

| 6-month follow-up (190±25 days) | Num. of patients | Incidence of stroke recurrence (%) | Much improvement (%) |
| --- | --- | --- | --- |
| Time from symptom onset to enrollment |  |  |  |
| ≤1year | 5 | 0 (0.0) | 5 (100.0) |
| >1 year | 1 | 0 (0.0) | 1 (100.0) |
| *p*-value | - | - | - |
| Modified Suzuki scoring |  |  |  |
| Stage I | 0 | - | - |
| Stage II | 0 | - | - |
| Stage III | 4 | 0 (0.0) | 4 (100.0) |
| Stage IV | 2 | 0 (0.0) | 2 (100.0) |
| *p*-value | - | - | - |
